# Prognostic Value of Complement Properdin in Cancer

**DOI:** 10.3389/fimmu.2020.614980

**Published:** 2021-01-19

**Authors:** Alessandro Mangogna, Praveen M. Varghese, Chiara Agostinis, Salman H. Alrokayan, Haseeb A. Khan, Cordula M. Stover, Beatrice Belmonte, Anna Martorana, Giuseppe Ricci, Roberta Bulla, Uday Kishore

**Affiliations:** ^1^Institute for Maternal and Child Health, IRCCS (Istituto di Ricovero e Cura a Carattere Scientifico) Burlo Garofolo, Trieste, Italy; ^2^Biosciences, College of Health, Medicine and Life Sciences, Brunel University London, Uxbridge, United Kingdom; ^3^School of Biosciences and Technology, Vellore Institute of Technology, Vellore, India; ^4^Department of Biochemistry, College of Science, King Saud University, Riyadh, Saudi Arabia; ^5^School of Biological Sciences, University of Leicester, Leicester, United Kingdom; ^6^Tumor Immunology Unit, Department of Health Promotion, Mother and Child Care, Internal Medicine and Medical Specialties, University of Palermo, Palermo, Italy; ^7^Department of Health Promotion, Mother and Child Care, Internal Medicine and Medical Specialties, University of Palermo, Palermo, Italy; ^8^Department of Medical, Surgical and Health Science, University of Trieste, Trieste, Italy; ^9^Department of Life Sciences, University of Trieste, Trieste, Italy

**Keywords:** properdin, innate immunity, bioinformatics, cancer, complement, prognosis

## Abstract

The complement system is readily triggered by the presence of damage-associated molecular patterns on the surface of tumor cells. The complement alternative pathway provides rapid amplification of the molecular stress signal, leading to complement cascade activation to deal with pathogens or malignant cells. Properdin is the only known positive regulator of the alternative pathway. In addition, properdin promotes the phagocytic uptake of apoptotic T cells by macrophages and dendritic cells without activating the complement system, thus, establishing its ability to recognize “altered-self”. Dysregulation of properdin has been implicated in substantial tissue damage in the host, and in some cases, chronic unresolved inflammation. A corollary of this may be the development of cancer. Hence, to establish a correlation between properdin presence/levels in normal and cancer tissues, we performed bioinformatics analysis, using Oncomine and UALCAN. Survival analyses were performed using UALCAN and PROGgeneV2 to assess if properdin can serve as a potential prognostic marker for human lung adenocarcinoma (LUAD), liver hepatocellular carcinoma (LIHC), cervical squamous cell carcinoma (CESC), and pancreatic adenocarcinoma (PAAD). We also analyzed levels of tumor-infiltrating immune cells using TIMER, a tool for characterizing immune cell composition in cancers. We found that in LUAD and LIHC, there was a lower expression of properdin in the tumors compared to normal tissues, while no significant difference was observed in CESC and PAAD. Survival analysis demonstrated a positive association between properdin mRNA expression and overall survival in all 4 types of cancers. TIMER analysis revealed that properdin expression correlated negatively with tumor purity and positively with levels of infiltrating B cells, cytotoxic CD8^+^ T cells, CD4^+^ helper T cells, macrophages, neutrophils and dendritic cells in LUAD, CESC and PAAD, and with levels of B cells, CD8^+^ T cells and dendritic cells in LIHC. Immunohistochemical analysis revealed that infiltrating immune cells were the most likely source of properdin in the tumor microenvironment. Thus, complement protein properdin shows promise as a prognostic marker in cancer and warrants further study.

## Introduction

Properdin, a ~50 kDa glycoprotein found at 4–25 μg/ml level in plasma, is the only known positive regulator of the complement system (1, 2). The complement system is an integral part of the innate immune system. It consists of three pathways, based on the recognition subcomponents as well as pathogen-associated molecular patterns (PAMPs) or danger-associated molecular patterns (DAMPs) on activators such as pathogens and IgG/IgM (3). The complement classical pathway is triggered by C1q interaction with IgG/IgM bound to target ligands; the lectin pathway, a homolog of the classical pathway, is triggered by the binding of mannan-binding lectin (MBL) to the carbohydrate patterns on the pathogen surface (4, 5). The complement alternative pathway is triggered by the spontaneous hydrolysis of complement protein C3 into C3(H_2_O) (6, 7), which in the presence of surface PAMPs, binds to the zymogen form of Factor B to form the fluid-phase C3 proconvertase, C3(H_2_O)B (8). Factor B is then cleaved by the serine protease, Factor D, into fragments Ba and Bb; Bb remains bound to C3(H_2_O) non-covalently producing C3(H_2_O)Bb, which is stabilized by binding to properdin yielding C3(H_2_O)BbP, the alternative pathway fluid-phase C3 convertase (2). C3(H_2_O)Bb further cleaves additional molecules of C3 into C3a and C3b (2); C3b gets deposited on the pathogen cell surface, where it associates with Factor B to generate C3bB, the membrane-bound C3 proconvertase (4). Factor D then cleaves Factor B in the C3bB complex and binds to properdin to generate the membrane-bound C3 convertase, C3bBbP (4), which cleaves several C3 molecules, leading to C3b deposition on the pathogen surface (9). C3b deposition is essential for opsonisation while the chemoattractant, C3a, helps mount an inflammatory response (9). Additionally, C3b also associates with C3bBb and properdin, forming the C5 convertase, C3bBbP3b (10) that cleaves C5 into C5a (a potent chemoattractant) and C5b. C5b sequentially binds C6, C7, C8 and multiple molecules of C9 to form the membrane attack complex (MAC) (10). The MAC disrupts the cell membrane of the pathogen causing cell lysis (11–13).

The alternative pathway is tightly regulated by the interactions of Factor H, C3b and properdin. In the presence of polyanionic markers that are present on “Self” cells, Factor H binds to their surface and dissociates Bb from the proconvertase (C3bBb and C3(H_2_O)Bb), thereby accelerating the decay of the convertases (2, 14). Structural analysis of properdin has revealed that it can interfere with this decay activity of Factor H (15). Factor H also prevents the formation of new convertases by playing a role as a cofactor for Factor I-mediated cleavage of C3b to “inactive” C3b (iC3b) and C3(H_2_O) to iC3(H_2_O) (4, 16). This cofactor activity has also been shown to be limited by properdin (17, 18). Properdin has also been shown to trigger the alternative pathway by directly recruiting C3b or C3(H_2_O) and Factor B (19). Properdin is found in circulation as cyclic dimers, trimers and tetramers in a 26:54:20 ratio *via* head-to-tail association of its monomers; it can interact with surface-bound iC3b, C3b and C3bBb with increasing efficiency (1, 17, 20).

Properdin is locally produced in secondary granules of stimulated peripheral blood neutrophils, dendritic cells and T cells (21–25). In addition to the C3 convertase-stabilizing function, properdin can also act as a humoral pattern recognition receptor (PRR) for ligands such as lipopolysaccharide, acetylated low-density lipoprotein, and zymosan (2). Surface bound properdin has also been shown to activate the alternative pathway by recruiting C3b and factor B to form C3bBbP (19). Properdin deficiency, an X-chromosomal recessive trait, is associated with ~250-fold more susceptibility to *Neisseria meningitidis* infection (26). In the case of non-infectious diseases such as rheumatoid arthritis, patients show decreased levels of properdin, C3 and C5, but high levels of Ba and C3d in the synovial fluid (27). Recent demonstration of properdin’s complement-independent functions as a PRR and modulator of pro-inflammatory immune response have suggested its important roles beyond the upregulation of the complement alternative pathway (28, 29).

Macrophages derived from properdin knockout mice showed a shift to M2 phenotype, with increased type II cellular immune responses and decreased MHC class II expression (30). The lack of properdin has also been shown to protect the host from an aggravated inflammatory response, increasing the survival of properdin gene knock-out mice compared to wild type mice (31–34). In colitis models, properdin deficiency intensified colonic injury, and more severe renal injury was reported in the presence of a dysfunctional Factor H and low properdin levels (35, 36). Properdin has also been reported to promote the uptake of apoptotic cells by phagocytes (37, 38).

Since properdin is a modulator of pro-inflammatory response and ~80%–90% of the terminal pathway is activated by the alternative pathway, the role of properdin in malignancy has been recently examined. Analysis of 13,023 genes for mutations between primary breast tumors and normal tissue revealed mutations at a significant frequency in the properdin (*CFP*) gene (39), hinting at mutants contribution to tumor progression (40). The tumorigenic consequences of dysregulated alternative pathway has been reaffirmed by a recent study that showed that aging factor H-deficient mice spontaneously develop hepatic tumor (~50% of male mice) (41); an increased expression of Factor H mRNA seemed associated with improved survival in hepatocellular carcinoma patients (41).

Here, we analyzed properdin expression and its correlation with prognosis using databases (Oncomine, UALCAN and PROGgeneV2) in lung adenocarcinoma (LUAD), liver hepatocellular carcinoma (LIHC), cervical squamous cell carcinoma (CESC), and pancreatic adenocarcinoma (PAAD), and interrogated if properdin could be an independent marker for diagnosis and a surrogate marker of severity and overall survival. Moreover, we investigated the correlation of properdin with tumor-infiltrating immune cells (TIICs) in all 4 types of cancers *via* Tumor IMmune Estimation Resource (TIMER) and performed immunohistochemical analysis to identify the most likely sources of properdin in the tumor microenvironment.

## Materials and Methods

### Gene Expression and Survival Analysis

The expression levels of *CFP* gene in different carcinomas were analyzed using Oncomine (www.oncomine.org) and UALCAN (http://ualcan.path.uab.edu). Oncomine is a cancer microarray database and web-based data mining platform from the genome-wide expression analyses. UALCAN, in addition to being a web resource for analyzing cancer transcriptome data, estimates the effects of gene expression levels on the patient survival (42–44). We compared the differences in mRNA levels between carcinomas and normal tissues. The mRNA expression levels in the neoplastic tissues, as compared to healthy tissues, were obtained as parameters of *p*-value < 0.05, fold change > 2, and gene ranking in the top 10%. Further information about the datasets can be found in **Table 1**.

**Table 1:** Characteristics of the datasets used in bioinformatics analysis.

Datasets	Study Description	Experiment Type
Hou Lung	91 non-small cell lung carcinoma and 65 adjacent normal lung samples were analyzed. Sample data includes age, sex, cancer sample site, and survival.	mRNA
Okayama Lung	226 lung adenocarcinoma samples and 20 normal lung tissues were analyzed. Sample data includes EGFR mutation, KRAS mutation, EML4-ALK gene fusion, stage, recurrence, survival status, and others.	mRNA
Bhattacharjee Lung	139 lung adenocarcinoma, 21 squamous cell lung carcinoma, 20 lung carcinoid tumor, six small cell lung carcinoma, and 17 normal lung samples were analyzed on Affymetrix U95A microarrays. Sample data includes type, age, M stage, max tumor percentage, N stage, primary/metastatic, recurrence, sex, site of metastasis, smoking rate (packs per year), stage, survival, and T stage.	mRNA
Su Lung	91 non-small cell lung carcinoma and 65 adjacent normal lung samples were analyzed. Sample data includes age, sex, cancer sample site, and survival.	mRNA
TCGA Lung	503 squamous cell lung carcinoma samples and 515 lung adenocarcinoma samples were analyzed on a custom Agilent microarray. Sample data includes age, TNM stage, sex, survival, smoking status, and others. This dataset is a combination of Lung Adenocarcinoma [LUAD] and Lung Squamous Cell Carcinoma [LUSC] data from the TCGA data portal and consists of Level 2 (processed) data.	mRNA
Roessler Liver	225 hepatocellular carcinoma and 220 normal liver samples were analyzed.	mRNA
Roessler 2 Liver	22 hepatocellular carcinoma, 19 normal liver, and two pooled normal liver tissue samples were analyzed.	mRNA
Wurmbach Liver	75 liver tissue samples were analyzed on Affymetrix U133 Plus 2.0 microarrays. This set of samples represents the stepwise carcinogenic process from preneoplastic lesions to hepatocellular carcinoma, in the following order: Normal Liver (n=10), Cirrhotic Liver (n=13), Dysplastic Liver (n=17), and Hepatocellular Carcinoma (n=35). All non-normal samples were hepatitis virus C (HCV) positive, but negative for other known causal agents of hepatocellular cancer. Sample data includes HCC stage, tumor size, differentiation, vascular invasion, and satellites.	mRNA
TCGA Liver	371 hepatocellular carcinoma, 56 paired normal blood, and 59 paired normal liver tissue samples were analyzed. Sample data includes sex, TNM stage, race/ethnicity, and others. This dataset consists of Level 3 data (segmented using CBS) from the TCGA data portal. The resulting segments were mapped to RefSeq gene coordinates as provided by UCSC. The samples were originally run on the Affymetrix SNP 6.0 platform.	mRNA
TCGA Cervix	305 cervical squamous cell carcinoma, 95 paired blood derived normal, and three paired normal cervix tissue samples were analyzed. Sample data includes age, TNM stage, grade, FIGO stage, survival and others. This dataset consists of Level 3 data (segmented using CBS) from the TCGA data portal. The resulting segments were mapped to RefSeq gene coordinates as provided by UCSC. The samples were originally run on the Affymetrix SNP 6.0 platform.	mRNA
TCGA Pancreas	178 pancreatic adenocarcinoma, 17 pancreatic ductal adenocarcinoma, one colloid carcinoma of the pancreas, 42 normal blood, and eight normal pancreas tissue samples were analyzed. This dataset consists of Level 3 data (segmented using CBS) from the TCGA data portal. The resulting segments were mapped to RefSeq gene coordinates as provided by UCSC. The samples were originally run on the Affymetrix SNP 6.0 platform.	mRNA

The prognostic significance of *CFP* mRNA expression with respect to survival in carcinomas was analyzed by UALCAN, using genomic data from “The Cancer Genome Atlas” (TCGA) to generate survival probability plots in the given carcinomas *via* Kaplan–Meier survival analysis (44). Additional survival analyses were performed with PROGgeneV2 (www.genomics.jefferson.edu/proggene/index.php) using data from TCGA (45, 46). Hazard ratio with a 95% confidence interval and log-rank *p*-value were also computed. In order to evaluate the prognostic effect of properdin, the four carcinomas we considered were LUAD, LIHC, CESC and PAAD.

### Protein Expression Analysis

The differential protein expression of properdin between carcinoma and normal tissues was analyzed *via* UALCAN, which allowed protein expression analysis using the Clinical Proteomic Tumor Analysis Consortium (CPTAC) Confirmatory/Discovery dataset (47).

### TIMER Database Analysis

TIMER is a comprehensive resource for systematic analysis of immune infiltrates across diverse cancer types (www.cistrome.shinyapps.io/timer) (48). TIMER applies a statistical method to infer the abundance of TIICs from the gene expression profiles using TCGA dataset (49). We analyzed, in LUAD, LIHC, CESC, PAAD, the correlation between *CFP* expression and the abundance of immune infiltrates, such as B cells, CD4^+^ T cells, CD8^+^ T cells, neutrophils, macrophages, and dendritic cells, *via* gene modules (50–52). The correlation module generated the expression scatter plots between the *CFP* gene and defined genes of TIICs in selected carcinomas, together with the Spearman’s correlation and the estimated statistical significance. *CFP* was plotted on the x-axis and the related marker genes were represented on the y-axis as genes of TIICs. The gene expression level was displayed with log_2_ RNA-Seq by Expectation-Maximization (RSEM).

### Immunohistochemical Analysis

Surgical normal tissue samples of lung, liver, uterine cervix and pancreas along with their malignant counterparts were selected for immunohistochemical analysis for properdin expression. Invasive malignant neoplasma specimens included the histotypes- LUAD, LIHC, CESC, and PAAD. The study was approved by the Institutional review board of the University of Palermo (09/2018). A specific informed consent was not required at the time of tissue sample collection for the immunohistochemical analysis of archival tissue sections since the patients were not identified and genetic analysis was not carried out. Immunohistochemistry (IHC) was performed using a polymer detection method. Briefly, tissue samples were fixed in 10% (v/v) neutral buffered formalin and then paraffin embedded. Tissue sections (4 μm thick) were deparaffinized and rehydrated. The antigen unmasking technique was carried out using Novocastra Epitope Retrieval Solutions, pH 9 (Leica Biosystems) in thermostatic bath at 98°C for 30 min. Sections were then brought to room temperature and washed in PBS. After neutralization of the endogenous peroxidase with 3% (v/v) H_2_O_2_ and Fc blocking by a specific protein block (Novocastra, Leica Biosystems), samples were incubated for 1 h at room temperature with mouse anti-human properdin (Clone C-4, 1:50, Santa Cruz) monoclonal antibody. Staining was revealed using Novolink Polymer Detection System (Novocastra, Leica Biosystems) and Romulin AEC Chromogen Kit (BioCare) as substrate chromogen, following the manufacturer**’**s instructions. Slides were counterstained with Harris Hematoxylin (BioOptica). Slides were analyzed under the Axio Scope A1 optical microscope (Zeiss) and microphotographs were collected through the Axiocam 503 color digital camera (Zeiss) using the Zen2 software.

### Statistical Analysis

Survival curves were generated by UALCAN and/or PROGgeneV2. All results were displayed with *p*-values from a log-rank test. *p*-values < 0.05 were considered significant (53, 54). Similarly, in the case of Oncomine, the program provided the statistical significance of the data (*p*-values). In TIMER, the correlation of gene expression was evaluated by Spearman’s correlation and statistical significance, and the strength of the correlation was determined using the following guide for the absolute value: 0.00-0.19: very weak; 0.20-0.39: weak; 0.40-0.59: moderate; 0.60-0.79: strong; and 0.80-1.0: very strong.

## Results

### Lower Properdin Expression Correlates With Poor Prognosis in Lung Adenocarcinoma and Vice Versa

**Figure 1:**
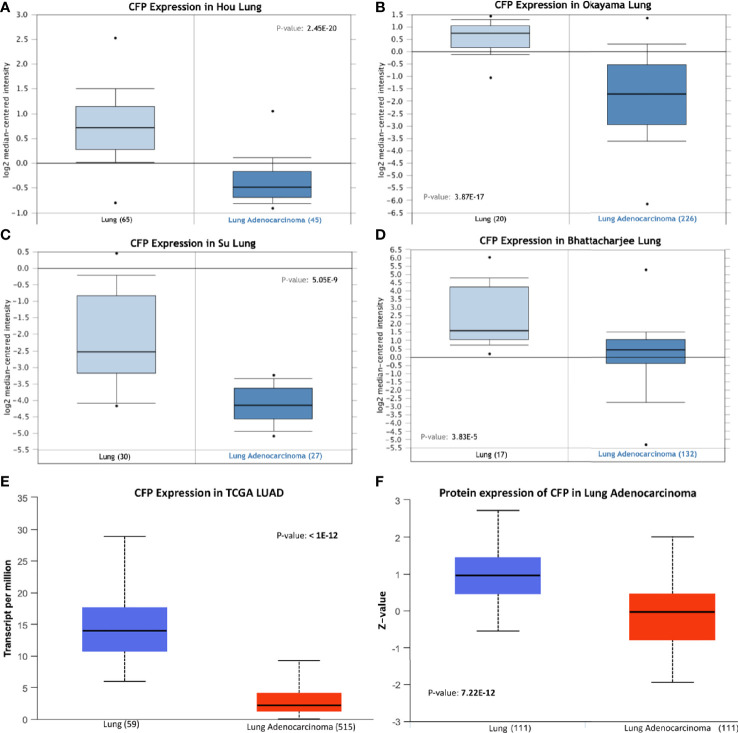
Properdin expression in lung adenocarcinoma. **(A–E)** *In silico* analysis of properdin (*CFP*) gene expression in lung adenocarcinoma (LUAD). *CFP* gene expression from Hou Lung **(A)**, Okayama Lung **(B)**, Su Lung **(C)**, and Bhattacharjee Lung **(D)** datasets plotted by Oncomine and The Cancer Genome Atlas (TCGA) Lung **(E)** dataset plotted by UALCAN tool revealed a decrease in properdin mRNA expression in the adenocarcinoma tissues when compared to the healthy lung tissues. *In silico* analysis of properdin (CFP) protein expression using CPTAC Confirmatory/Discovery **(F)** dataset analyzed using UALCAN confirmed higher levels of properdin in healthy tissues compared to the cancerous tissues, similar to the gene expression studies.

**Figure 2:**
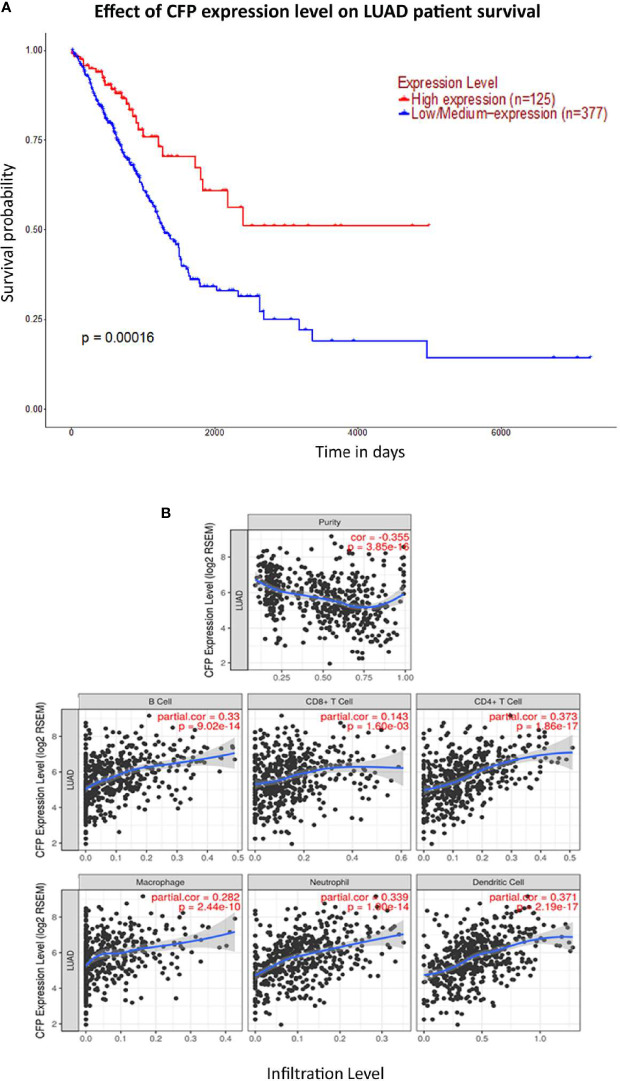
Prognostic value of properdin in lung adenocarcinoma. **(A)** An association between *CFP* expression and overall survival. Overall Survival (OS) of lung cancer patients expressing high [red] (n=125) and low/medium [blue] (n=377) levels of properdin over a period of ~7,000 days (~19 years) analyzed with Kaplan–Meier survival. Analysis using The Cancer Genome Atlas (TCGA) Lung dataset by UALCAN revealed that patients with high levels of properdin expression have had a significant improvement in survival compared to their low/medium expression counterparts. **(B)** The correlation of *CFP* gene expression with immune infiltration level in lung adenocarcinoma. Levels of tumor-infiltrating immune cells in the lung adenocarcinoma were analyzed from TCGA Lung dataset using TIMER. *CFP* expression was found to be significantly and negatively related to tumor purity. Significant positive correlations with infiltrating levels of B cells, CD8^+^ T cells, CD4^+^ T cells, macrophages, neutrophils and dendritic cells were observed with CFP expression. Genes highly expressed in the tumor microenvironment are expected to have negative associations with tumor purity, while the opposite is likely to be true for genes highly expressed in the tumor cells.

We initially evaluated the differences in the mRNA level of properdin in LUAD as compared to normal lung tissue, using the Oncomine platform *via* Hou’s, Okayama’s, Su’s and Bhattacharjee’s datasets (**Table 1**). We noted a significantly lower properdin mRNA expression in LUAD in comparison with healthy lung tissue counterpart (*p* < 0.05) (**Figures 1A–D**). Similar result was revealed by UALCAN tool analysis using the TCGA dataset (*p* < 0.05) (**Figure 1E**). UALCAN proteomics analysis also validated lower expression of properdin in LUAD, as compared to the corresponding healthy tissue (*p* < 0.05) (**Figure 1F**). As shown in **Figure 2A**, properdin mRNA expression positively correlated with the overall survival (OS) rate of LUAD patients (*p* < 0.05). Based on the TIMER analysis, *CFP* expression showed a significantly weak negative correlation with tumor purity (*p* < 0.05). It revealed a partial but significant and positive correlation with infiltrating levels of B cells, CD8^+^ T cells, CD4^+^ T cells, macrophages, neutrophils and dendritic cells (*p* < 0.05) (**Figure 2B**). The strength of the correlation was very weak for CD8^+^ T cells, whereas it was relatively weaker for B cells, CD4^+^ T cells, macrophages, neutrophils and dendritic cells.

**Figure 3:**
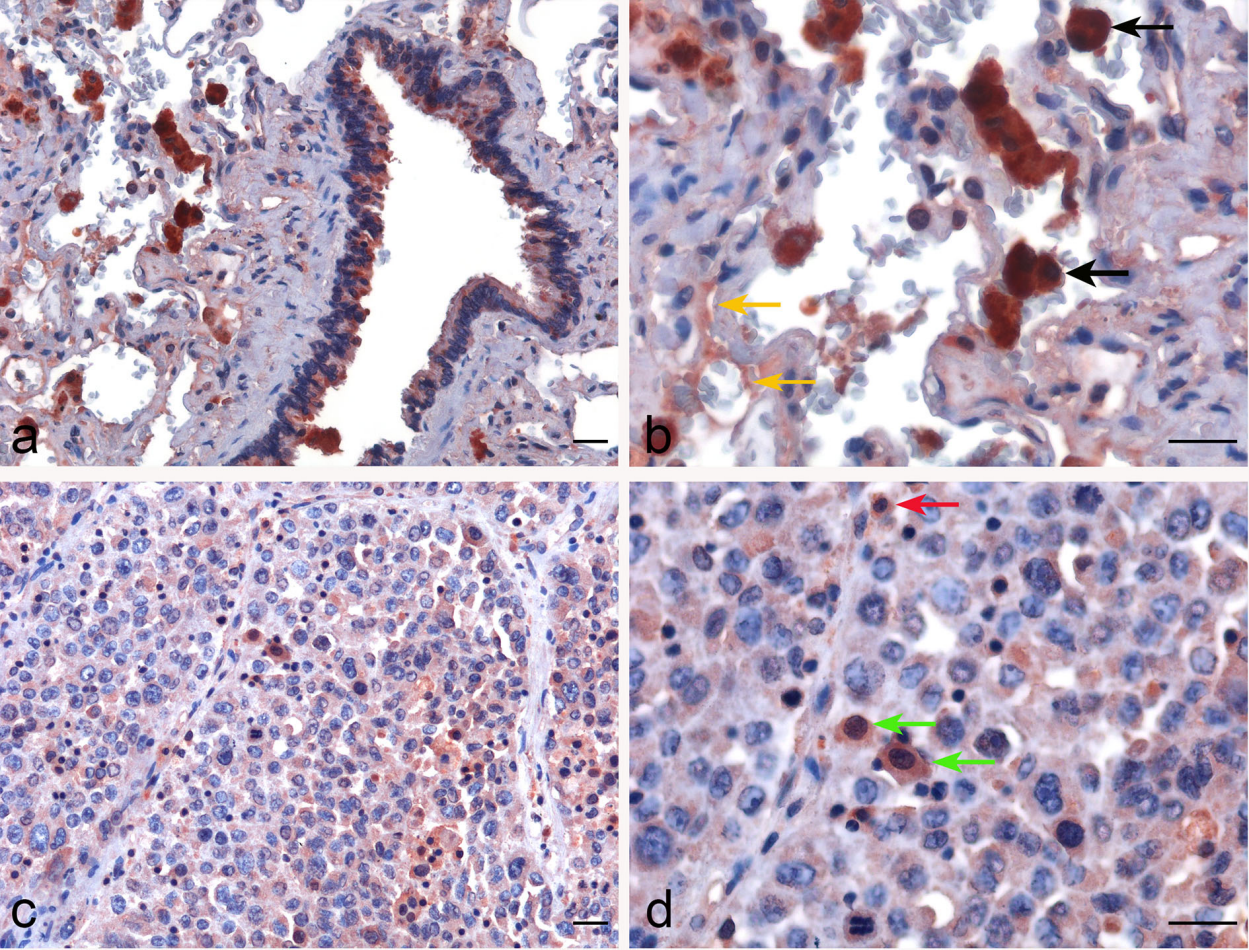
Immunohistochemical evaluation of properdin in healthy lung and lung adenocarcinoma tissues. Representative microphotographs pertaining to properdin expression in the alveolar macrophages (black arrows) and the endothelial cells (yellow arrows) in healthy pulmonary parenchyma **(A, B)**. Representative microphotographs of properdin staining detailing the presence of macrophages (green arrows) and granulocytes (red arrows) populating tumor infiltrates in lung adenocarcinoma (LUAD) **(C, D)**. Magnification 200 x **(A–C)**, 400 x **(B, D)**; scale bars 50 μm.

In lung IHC staining, with membrane as well as cytoplasmic labeling, properdin was found to be expressed mainly by the alveolar macrophages in a diffuse pattern; endothelial cells labeled mildly (**Figures 3A, B**). In LUAD, we observed properdin expression within the tumor-associated immune infiltrates (**Figures 3C, D**). Properdin positive cells were mainly represented by macrophages and somewhat minimally by granulocytes, present within the tumoral nests as well as in the intratumoral stromal microenvironment. Moreover, the density of properdin positive cells showed a variable distribution in the neoplastic tissue; in fact, it was higher in the inter-tumoral stroma compared to the one detected in the neoplastic proliferation. Thus, the immunohistochemical assessment confirmed that the immune cells were the main source of properdin.

### Properdin Expression in Liver Hepatocellular Carcinoma Correlates Positively With Overall Survival and Immune Infiltration in the Tumor Microenvironment

**Figure 4:**
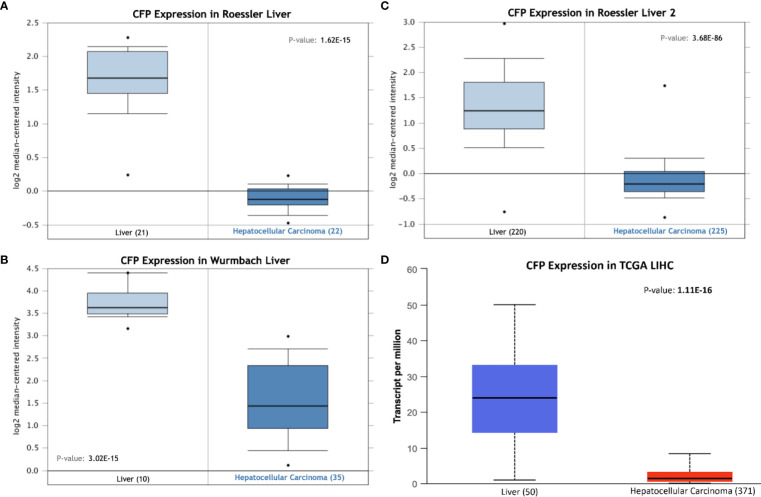
Properdin expression in liver hepatocellular carcinoma. *In silico* analysis of properdin (*CFP*) gene expression in liver hepatocellular carcinoma (LIHC). *CFP* gene expression from Roessler **(A)**, Roessler 2 **(B)**, Wurmbach **(C)** Liver datasets plotted by Oncomine and The Cancer Genome Atlas (TCGA) Liver **(D)** dataset plotted by UALCAN tool revealed a decrease in properdin mRNA expression in the adenocarcinoma tissues, when compared to the healthy liver tissues.

**Figure 5:**
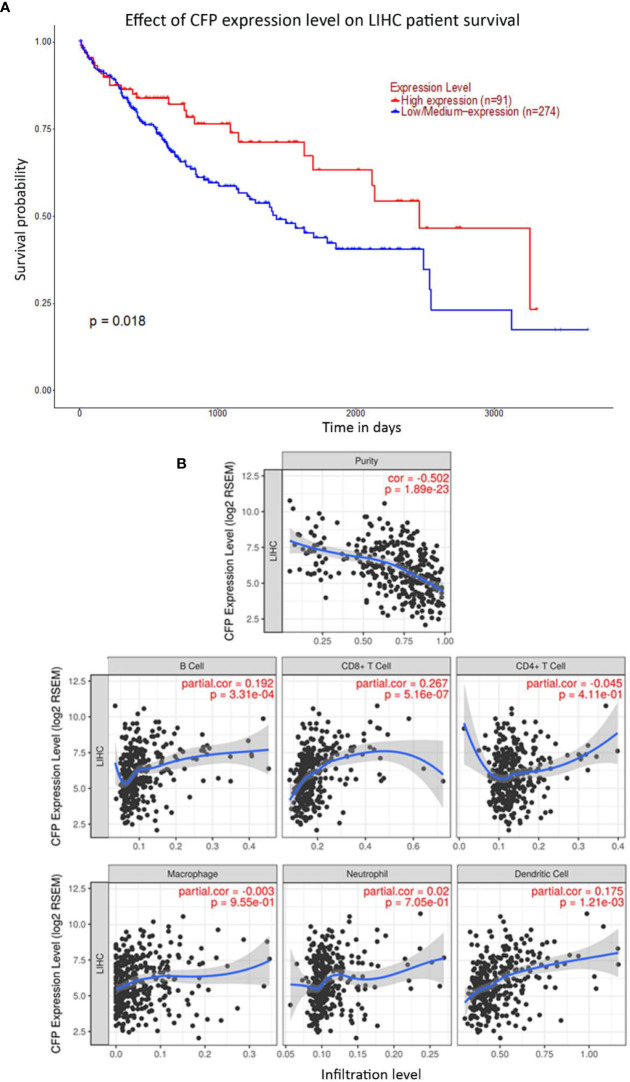
Prognostic effect of properdin on liver hepatocellular carcinoma. **(A)** The association between *CFP* expression and overall survival. Overall Survival (OS) of liver cancer patients expressing high [red] (n=91) and low/medium [blue] (n=274) levels of properdin over a period of ~4,000 days (~11 years) analyzed with Kaplan–Meier survival analysis using The Cancer Genome Atlas (TCGA) Liver dataset by UALCAN shows patients with high levels of properdin expression shows a significant improvement in survival compared to their low/medium expression counterparts. **(B)** The correlation of *CFP* gene expression with immune infiltration level in liver hepatocellular carcinoma. Levels of tumor-infiltrating immune cells in liver hepatocellular carcinoma were analyzed from TCGA Liver dataset using TIMER. CFP expression was significantly negatively related to tumor purity. It showed significant positive correlations with infiltrating levels of B cells, CD8^+^ T cells and dendritic cells. No significant correlation was evident with the infiltrating levels of CD4^+^ T cells, macrophages and neutrophils.

Bioinformatics analysis of properdin mRNA expression was performed in the context of the LIHC using the Roessler, Roessler 2, Wurmbach and TCGA dataset. Similar to LUAD, a lower expression level of properdin was detected as compared to normal liver tissue (*p* < 0.05) (**Figure 4**). According to UALCAN tool, properdin mRNA expression positively associated with an OS rate in LIHC patients (*p* < 0.05) (**Figure 5A**). A significant and moderate negative correlation between properdin expression and tumor purity was also observed (*p* < 0.05) (**Figure 5B**). It showed partial but significant and positive correlations with infiltrating levels of B cells, CD8^+^ T cells and dendritic cells (*p* < 0.05). The strength of the correlation was very weak for B cells and dendritic cells, whereas it was weak for CD8^+^ T cells. No significant correlation between the infiltrating levels of CD4^+^ T cells, macrophages and neutrophils and CFP expression was noted in LIHC.

**Figure 6:**
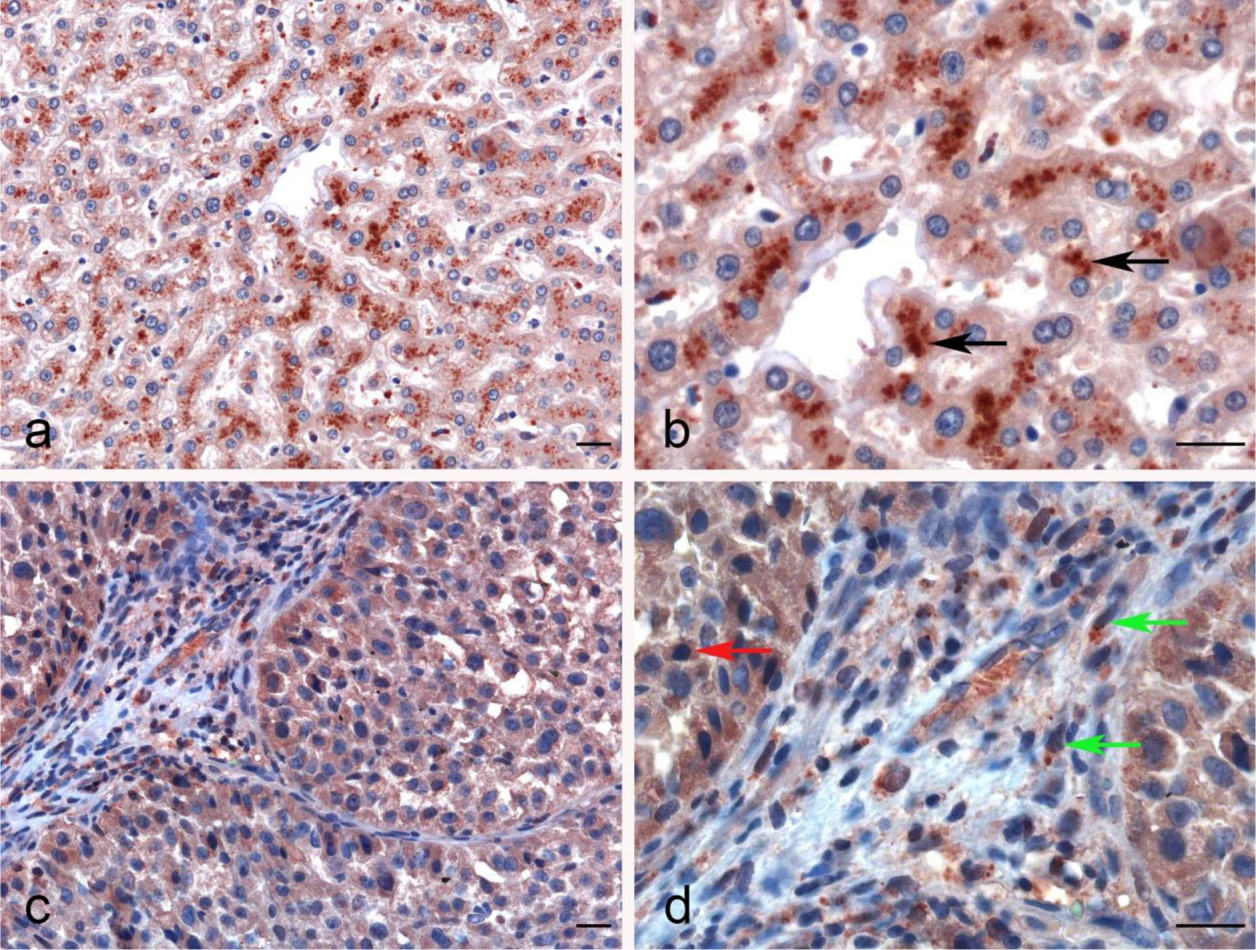
Immunohistochemical analysis of properdin in normal liver and hepatocellular carcinoma tissues. Properdin immunohistochemistry (IHC) staining showing the strong cytoplasmic expression by the hepatocytes in normal liver (black arrows) **(A, B)**. Representative microphotographs detailing the macrophage-associated tumor (green arrows) and a few neoplastic subclones (red arrow) expressing properdin in liver hepatocellular carcinoma (LIHC) **(C, D)**. Magnification 200 x **(A–C)**, 400 x **(B, D)**; scale bars 50 μm.

The IHC staining of liver parenchyma for properdin highlighted a variable pattern: ~30%–40% of hepatocytes exhibited cytoplasmic expression of properdin. A few immune infiltrates normally present within the portal space and in the portal stroma also showed properdin positivity (**Figures 6A, B**). In LIHC, immunohistochemical staining revealed properdin expression in the TIICs within the stromal microenvironment as well as a few neoplastic components (~2% of total tumor cells) (**Figures 6C, D**).

### Properdin Is Expressed in Cervical Squamous Cell Carcinoma and Normal Cervical Tissues at Similar Levels

**Figure 7:**
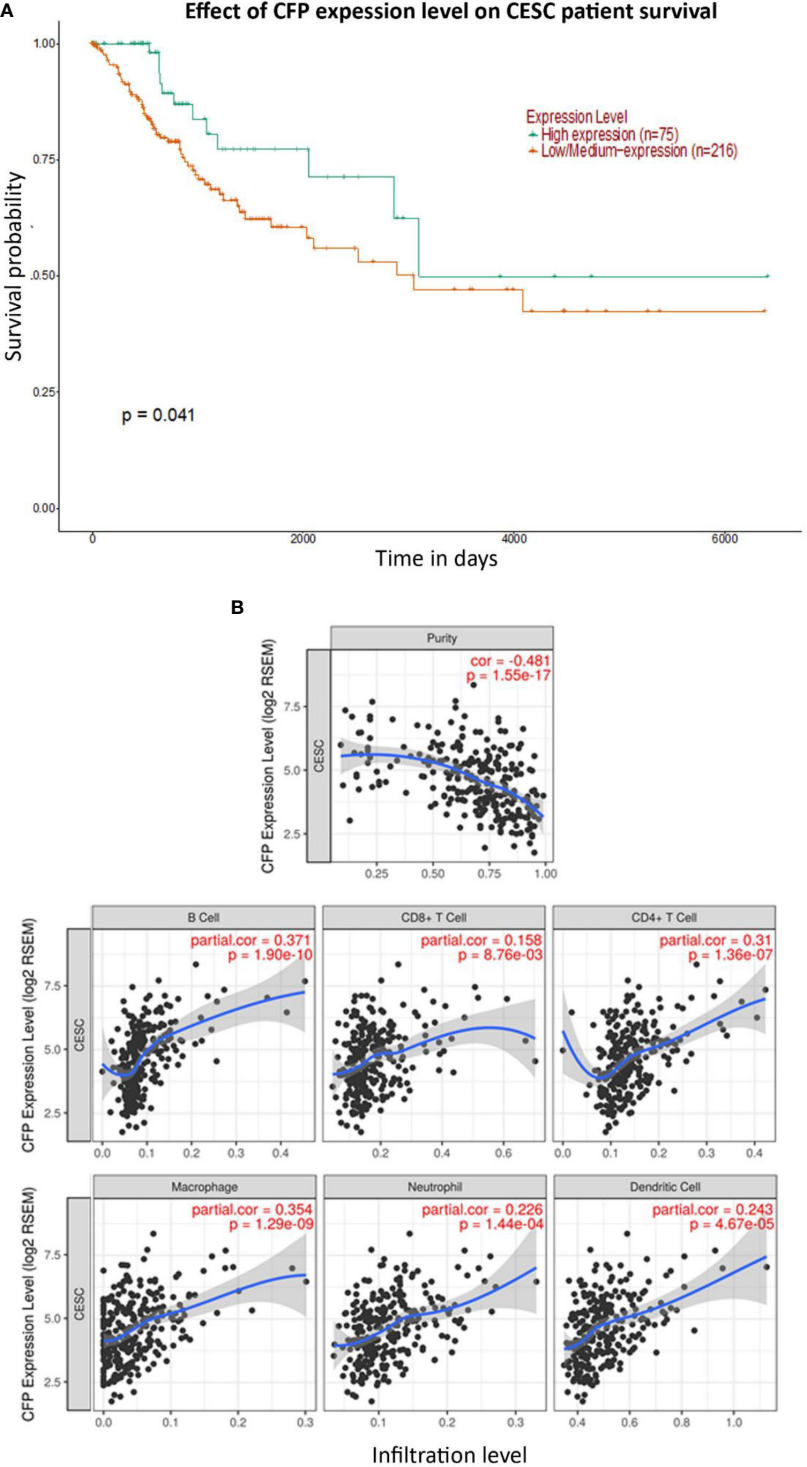
Prognostic effect of properdin expression in cervical squamous cell carcinoma. **(A)** The association between *CFP* expression and overall survival. Overall Survival (OS) of cervical squamous cell carcinoma (CESC) patients expressing high [green] (n=75) and low/medium [orange] (n=216) levels of properdin over a period of ~7,000 days (~19 years), analyzed with Kaplan–Meier survival analysis using The Cancer Genome Atlas (TCGA) Cervix dataset by UALCAN, revealed that patients with high levels of properdin expression had a significant improvement in survival compared to their low/medium expression counterparts. **(B)** The correlation of *CFP* gene expression with immune infiltration level in cervical squamous cell carcinoma. Levels of tumor-infiltrating immune cells in cervical squamous cell carcinoma (CESC) were analyzed from TCGA Cervix dataset using TIMER. CFP expression was significantly and negatively related to tumor purity. It had a significant positive correlation with infiltrating levels of B cells, CD8^+^ T cells, CD4^+^ T cells, macrophages, neutrophils and dendritic cells. Genes highly expressed in the microenvironment are expected to have negative association with tumor purity, while the opposite is the case for genes highly expressed in the tumor cells.

**Figure 8:**
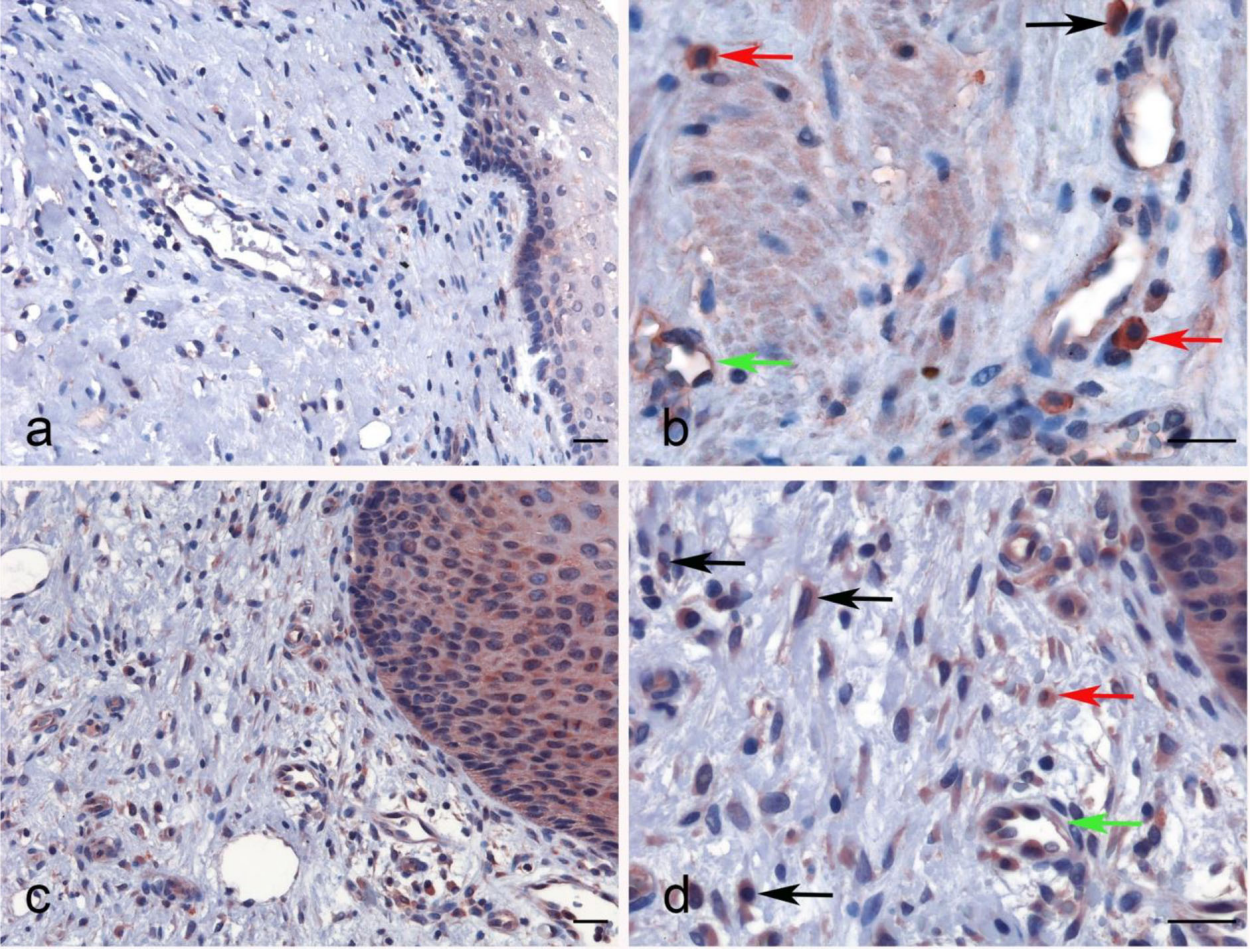
Immunohistochemical analysis for properdin expression in uterine cervix and cervical squamous cell carcinoma. Representative images of macrophages (black arrow), lymphoid cells (red arrow) and also the endothelial cells of vessels within the chorion (green arrow) expressing properdin in the healthy uterine cervix **(A, B)** and CECS **(C, D)**. Magnification 200 x **(A–C)**, 400 x **(B, D)**; scale bars 50 μm.

No significant difference regarding properdin expression level in CECS was detected as compared to normal cervical tissues using different datasets (Data Not Shown). However, among CECS patients, a higher expression of properdin positively correlated with an OS (*p* < 0.05) (**Figure 7A**). According to the TIMER tool, *CFP* gene expression moderately, but significantly and negatively, correlated with the tumor purity (*p* < 0.05). It revealed a partial but significant and positive correlation with the levels of infiltrated B cells, CD8^+^ T cells, CD4^+^ T cells, macrophages, neutrophils and dendritic cells (*p* < 0.05) (**Figure 7B**). The strength of the correlation was very weak for CD8^+^ T cells, whereas it was weak for B cells, CD4^+^ T cells, macrophages, neutrophils and dendritic cells. In the uterine cervical tissue, immunohistochemical assessment for the presence of properdin protein revealed a high expression in both the normal as well as the tumoral tissues (**Figure 8**). Properdin appeared to be diffusely expressed not only within the infiltrating cells (macrophages and few cells of lymphoid morphology), but also within the endothelial cells of small vessels located in the subepithelial connective tissue.

### Properdin in Pancreatic Adenocarcinoma

**Figure 9:**
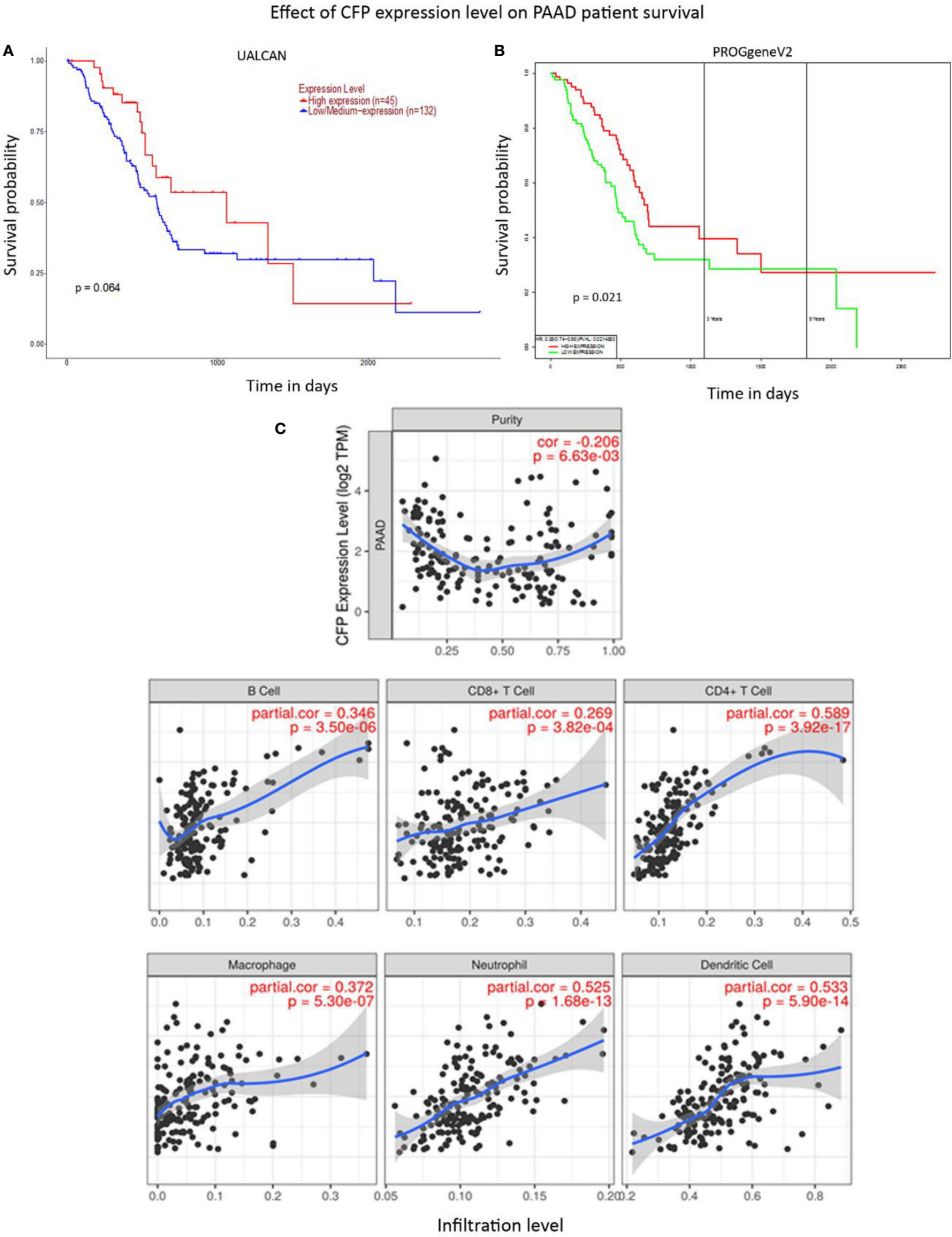
Prognostic significance of properdin in pancreatic adenocarcinoma. **(A)** The association between CFP expression and overall survival. Overall Survival (OS) of pancreatic adenocarcinoma (PAAD) patients expressing high [red] (n=45) and low/medium [blue] (n=132) levels of properdin in the tissues over a period of ~3,000 days (~8 years) analyzed with Kaplan–Meier survival analysis using The Cancer Genome Atlas (TCGA). Pancreas dataset by UALCAN showed no correlation between properdin expression levels and OS. **(B)** PROGgeneV2 analyzed OS association with CFP expression in PAAD patient tissues expressing high [red] (n=85) and low/medium [green] (n=85) levels of properdin over a period of ~3,000 days (~8 years) using TCGA Pancreas dataset and showed significant improvement in survival in patients expressing higher levels of properdin compared to their low/medium expressing counterparts. The variation in OS results can be attributed to the way the tools define their patient cohorts. **(C)** The correlation between CFP gene expression and immune cell infiltration level in pancreatic adenocarcinoma. Levels of tumor-infiltrating immune cells in PAAD were analyzed from TCGA Pancreas dataset using TIMER. CFP expression appeared to exhibit a significantly negative correlation with tumor purity. There appeared to be a significant positive correlation with infiltrating levels of B cells, CD8^+^ T cells, CD4^+^ T cells, macrophages, neutrophils and dendritic cells. Genes highly expressed in the tumor microenvironment are expected to have negative association with tumor purity, while the opposite is likely to be case for genes highly expressed in the tumor cells.

**Figure 10:**
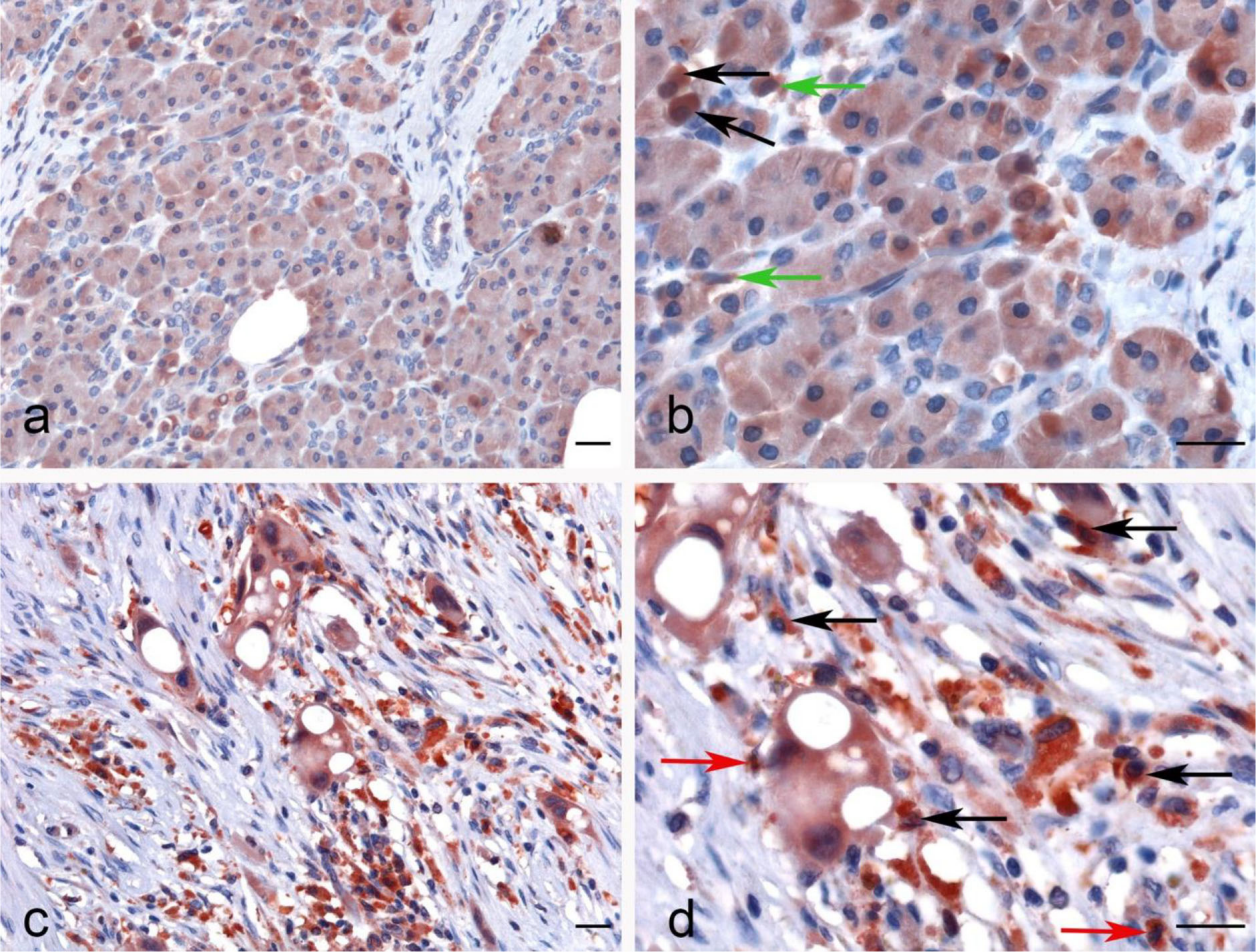
Immunohistochemical analysis of properdin expression in pancreatic parenchyma and pancreatic ductal adenocarcinoma. Representative IHC images for properdin displaying the expression by acinar cells (black arrows) and also by few macrophages present in the connective tissue (green arrows) in normal pancreatic parenchyma **(A, B)**. In PAAD, the tumor-associated immune infiltrates, consisting of macrophages (black arrow) and granulocytes (red arrows), seemed to express properdin strongly **(C, D)**. Magnification 200 x **(A–C)**, 400 x **(B, D)**; scale bars 50 μm.

No significant difference regarding properdin expression level in PAAD was detected as compared to normal pancreas using various datasets (Data Not Shown). According to UALCAN tool, no correlation between properdin mRNA expression and OS rate was observed in patients with PAAD (**Figure 9A**). However, when we used PROGgeneV2 tool, properdin mRNA expression was found to be positively associated with an OS rate in PAAD patients (*p* < 0.05) (**Figure 9B**). With respect to immune infiltration, CFP gene expression showed a partial but significant and negative relationship with the tumor purity (*p* < 0.05). This means that properdin is unlikely to be expressed by tumor cells but by the immune infiltrates. It suggested significant positive partial correlations with infiltrating levels of B cells, CD8^+^ T cells, CD4^+^ T cells, macrophages, neutrophils and dendritic cells (*p* < 0.05) (**Figure 9C**). The strength of the correlation was weak for B cells, CD8^+^ T cells and macrophages, whereas it was found to be moderate for CD4^+^ T cells, neutrophils and dendritic cells. IHC staining of the healthy pancreatic tissue revealed that ~10% of acinar cells of the exocrine part of pancreas had a high expression of properdin (strong cytoplasmic labeling) (**Figure 10A**). The immune infiltrate, consisting of scant macrophages present in the connective tissue contexture also stained for properdin (**Figure 10B**). A marked expression of properdin by the infiltrating immune cells, mostly consisting of macrophages and granulocytes, was evident; however, we observed no properdin expression in the neoplastic subclones (**Figures 10C, D**).

## Discussion

The innate immunity plays a critical role in the protection against cancer. The complement system is an important arsenal of the innate immune system against pathogens as well as cancer. The complement system is initiated in response to the tumor-associated antigens, and leads to increased deposition of complement activation fragments on the surface of the tumor. C1q, for instance, has been shown to bind to phospholipids of lung tumor cell lines and evidence of activation of the classical pathway in various cancer such as lung cancer, thyroid cancer, astrocytoma, lymphoma, leukemia, and oropharyngeal cancer have been reported (55, 56). C1q has recently been shown to be involved in a range of pathophysiological functions that are not dependent on complement activation. In fact, it is expressed in the microenvironment of various types of human tumors, where it can exert a protective or a harmful effect on cancer progression (57–60). Similarly, in cases of acute lymphoblastic leukemia, Burkitt lymphoma or multiple myeloma, dysregulation of the complement alternative pathway has been reported (61). Exaggerated or aberrant complement activation in the tumor microenvironment has been shown to increase inflammation/infiltration *via* C5a. Anti-tumor immune responses are stifled by the C5a-driven recruitment of myeloid-derived suppressor cells, which support tumorigenesis by upregulating anti-inflammatory (inhibitory) molecules such as Programmed Death-Ligand 1 (PD-L1), transforming growth factor-β (TGF-β) and IL-10 (62–64). C3a has also been implicated in tumorigenesis; C3aR^−/−^ mice show improved disease outcome in melanoma (65). Factor H gene-deficient mice appear to develop spontaneous hepatocellular carcinoma with aging (41). An *in silico* analysis appeared to suggest a protective role of properdin in breast cancer by interacting with glycosaminoglycan (GAG) structures on the tumor cell surface (39, 41, 66).

In the current study, we analyzed the prognostic value of properdin, the positive regulator of the complement alternative pathway, in four types of cancer, namely LUAD, LIHC, CESC, and PAAD. The choice of the four tumors was made based on the prognostic effect of properdin, which was assessed *via* UALCAN using TCGA RNA-sequencing and patients’ clinical data from 33 different cancer types, including several metastatic tumors. Of the 33 cancers, properdin had a significant prognostic effect in only 4 neoplasms. Subsequently, *CFP* gene expression in various neoplastic tissues was analyzed using Oncomine and UALCAN, and compared with their healthy counterparts. Lower levels of CFP expression were observed in both LUAD and LIHC tissues compared to their respective normal tissues. No significant difference was observed in the expression levels in the case of CESC and PAAD. Proteomic analysis of properdin levels in LUAD was also analyzed by UALCAN and found to be lower in the neoplastic tissue compared to the healthy tissues. However, currently, proteomic analysis of the other three cancers are not available on UALCAN. Kaplan–Meier survival analysis of LUAD, LIHC and CESC by UALCAN revealed that properdin mRNA expression positively correlated with an OS rate of the patients. In the case of PAAC, UALCAN survival analysis did not find any correlation between the mRNA expression levels and OS; however, analysis by PROGgeneV2 revealed a positive correlation between mRNA expression levels and OS. This variation can be attributed to the difference in the way the tools define patient cohorts, as both tools use the same TCGA Pancreas dataset. UALCAN divides patients into a high expression of properdin versus low/medium expression. PROGgeneV2 divides patients at the median of gene expression between the ones with low expression and those with high expression. This is a limitation of the study involving bioinformatics tools that can be used to evaluate the prognostic implications of genes in various cancers. Thus, using the same cohort of patients (for example, TCGA Pancreas dataset), the results on the prognostic effect can differ, depending on whether the court is divided by the median of expression or not.

It is highly plausible that the expression of the *CFP* gene during tumorigenesis may lead to properdin export to the cell surface (66), which may then promote phagocytosis by macrophages and dendritic cells (37, 38), in addition to recruiting immune cells. Thus, the neoplastic cells have evolved to express low levels of properdin, and hence, higher expression of the protein leads to increased overall survival. Since TIICs are independent predictors of sentinel lymph node status and survival in cancer (67, 68), we investigated whether *CFP* expression correlated with immune infiltration levels in various cancer types. Tumor purity is defined as the proportion of cancer cells in the tumor tissue or the percentage of cancer cells in a solid tumor sample. *CFP* expression negatively correlated with the tumor purity in all cancers. This suggests that the expression of properdin likely occurs by the immune infiltrates and not by the actual neoplastic cells. Infiltrating levels of B cells, CD8^+^ T cells, CD4^+^ T cells, macrophages, neutrophils and dendritic cells positively correlated in the case of LUAD, CESC and PAAD. In the case of LIHC, a positive correlation with infiltrating levels of B cells, CD8^+^ T cells and dendritic cells was observed, while no significant correlation was observed between the expression of *CFP* and the levels of infiltrating CD4^+^ T cells, macrophages and neutrophils. These findings suggest that *CFP* plays a specific role in immune infiltration in our carcinomas, in particular as regard CD4^+^ T cells, neutrophils and dendritic cells. Therefore, *CFP* could exert an important function in recruitment and regulation of immune infiltrating cells in several carcinomas.

Interestingly, preliminary studies conducted in properdin knock-out mice showed a difference in chemokine levels and cell mobility in a tumor model (CM Stover, unpublished Data). The role of immune cell infiltration in the tumor microenvironment and its consequences on tumor development is an intense area of research: immune infiltration and cell phenotype polarization can be a double-edged sword. It can promote or impede tumor growth, and hence, overall survival. Given a positive correlation with immune infiltration, the properdin expression levels reflect the presence of immune cells that are less immunosuppressive but can engage in the anti-tumor response. Another possibility is that properdin acts as a balancing factor between macrophage polarization between pro-inflammatory M1 and anti-inflammatory M2 phenotypes (30). NK cells, a key player in the anti-tumor immunity, have recently been shown to bind properdin through NKp46 receptor (69). However, how properdin can impact on NK cell-mediated properties in the tumor microenvironment is unclear.

Anti-tumor immunity has been recently categorized into mainly three phenotypes: immune-desert (immune ignorance), immune-excluded (inhibitory chemokine/soluble factor-mediated), and inflamed (where infiltration is immunosuppressive and/or dysregulated) (70). The immune cell infiltration here includes regulatory T cells, myeloid-derived suppressor cells, suppressor B cells and fibroblasts. Tumor-infiltrating CD8^+^ T lymphocytes can also be aberrantly active and immunologically exhausted. In a recent study, Troiano et al. identified a specific subgroup of squamous cell carcinoma of the oral tongue with poor prognosis based on the density of tumor‐infiltrating lymphocytes and localization (71). Thus, the tumor immune phenotyping can be a better approach to stratify patients and decide on precision medicine.

Understanding how the level of properdin expressed in the tumor-infiltrating immune cells alters the tumor microenvironment and manipulates immune cells versus tumor cells should be of great relevance for immunotherapeutic development. Hence, further study to examine the differential expression and distribution of properdin along with immune cells in the neoplastic tissues is urgently required. As properdin has also been shown to function in a complement-independent manner (27, 28, 37), experiments in the absence of the complement system and with an individual pathway blocked must also be undertaken. In conclusion, our *in-silico* analysis highlights a possible role of properdin as a novel marker for tumor prognosis and increased survival in a range of cancers.

## Data Availability Statement

The original contributions presented in the study are included in the article/supplementary materials; further inquiries can be directed to the corresponding authors.

## Author Contributions

AlM, PV, BB, AnM, CA, and GR generated and analyzed the data. SA, HK, and CS interpreted and contributed to the draft. RB and UK led the work. AnM and PV did the first draft. RB and UK finalized the manuscript. All authors contributed to the article and approved the submitted version.

## Conflict of Interest

The authors declare that the research was conducted in the absence of any commercial or financial relationships that could be construed as a potential conflict of interest.
